# Perceived severity and parental distress after positive expanded newborn screening: parent–clinician concordance and dyadic processes

**DOI:** 10.1007/s00431-026-06983-7

**Published:** 2026-04-29

**Authors:** Marco Bani, Selena Russo, Serena Gasperini, Viola Crescitelli, Francesca Menni, Francesca Furlan, Francesco Tagliaferri, Graziella Cefalo, Sabrina Paci, Giuseppe Banderali, Paola Marchisio, Adriana Balduzzi, Maria Grazia Strepparava

**Affiliations:** 1 School of Medicine and Surgery, University of Milano-Bicocca, Via Cadore 48, 20900 Monza, Italy; 2 Department of Pediatrics, Fondazione IRCCS San Gerardo Dei Tintori, Monza, Italy; 3 Regional Clinical Center for Expanded Newborn Screening, Fondazione IRCCS Ca’ Granda Ospedale Maggiore Policlinico, Milan, Italy; 4 Department of Clinical Sciences and Community Health, University of Milan, Dipartimento Di Eccellenza, Milan, 2023-2027 Italy; 5 Department of Woman and Child, Pediatric Unit, ASST Santi Paolo e Carlo, Milan, Italy; 6 Clinical Psychology Unit, Fondazione IRCCS San Gerardo dei Tintori, Monza, Italy

**Keywords:** Communication of positivity, Concordance, Expanded newborn screening, Metabolic diseases, Parental distress, Severity

## Abstract

Receiving a positive expanded newborn screening result is highly stressful for parents. This study aimed to examine parent–clinician concordance in perceived severity, to investigate its association with parental emotional distress at baseline and six months after communication, and to explore the interdependence between perceived severity and perceived control within parental dyads. A total of. 171 mothers and 168 fathers reported parental distress and completed the Impact of Event Scale–Revised and the Emotion Thermometers. Perceived severity and control were measured using single-item Likert scales reflecting subjective appraisals at the time of disclosure. For 147 parents (74 mothers and 73 fathers), a clinician-rated severity score was available. An Actor–Partner Interdependence Model with indistinguishable dyads was applied to 115 parental couples. Most parents (61.9%) rated their child’s condition as more severe than the clinician. At baseline, higher parental severity ratings relative to clinicians were associated with higher distress and post-traumatic symptoms. No differences were observed at 6 months. Higher perceived severity in one parent was associated with lower perceived control in the partner (β = − 0.207, *p* = .004).

*Conclusion*: Differences between parental and clinician perceptions of severity are common following positive ENBS communication and are associated with elevated early parental distress. Psychosocial support may benefit from adopting a dyadic perspective, acknowledging that each parent’s appraisal of the situation can shape the partner’s adjustment.

**Table:** 

**What is Known:** • *Receiving communication of positivity at the ENBS can be highly stressful for parents.* • *Parents’ perceptions of disease severity play an important role in their psychological adjustment.*	
**What is New:** • *Parent–clinician misalignment in perceived severity is frequent after positive ENBS communication and is associated with higher early parental distress.* • *Parental perceptions of severity and control show significant dyadic interdependence within couples, high lighting parental adjustment as a relational process.*

**What is Known:**

• *Receiving communication of positivity at the ENBS can be highly stressful for parents.*

• *Parents’ perceptions of disease severity play an important role in their psychological adjustment.*

**What is New:**

• *Parent–clinician misalignment in perceived severity is frequent after positive ENBS communication and is associated with higher early parental distress.*

• *Parental perceptions of severity and control show significant dyadic interdependence within couples, high lighting parental adjustment as a relational process.*

## Introduction

Although ENBS aims to promote early detection and improved clinical outcomes, the initial disclosure of a suspected condition often occurs when the infant is asymptomatic, and the diagnostic picture remains uncertain. The communication of a positive result from neonatal screening for metabolic diseases can significantly affect family well-being, often generating considerable emotional distress and uncertainty while awaiting confirmatory diagnostic tests [1–6]. In this context, parents are abruptly confronted with the possibility of a serious health threat, frequently before confirmatory testing is completed.

Previous research has consistently documented high levels of parental distress, anxiety, and post-traumatic symptoms following the communication of a positive screening result, even in cases later classified as false positives or variants of uncertain significance [1–6]. Parental adjustment appears to be influenced not only by the objective diagnostic outcome but also by how the information is communicated and interpreted [7–9].

Within stress appraisal models and illness representation frameworks, emotional responses to health-related threats are shaped by individuals’ subjective appraisals of the illness, particularly perceptions of its severity and controllability [10, 11].

According to cognitive models of stress and illness representation, perceived severity reflects the anticipated seriousness and consequences of the condition, whereas perceived control refers to the extent to which individuals believe they can manage or influence the situation. Both dimensions are central to psychological adjustment: higher perceived severity and lower perceived control are consistently associated with increased distress in medical contexts [7, 8, 12]. In the newborn screening setting—characterized by uncertainty, limited experiential knowledge, and reliance on professional guidance—parents’ appraisals are likely to be strongly influenced by the clinical encounter in which the result is communicated. Furthermore, families’ perception of disease severity can influence their ability to tolerate uncertainty, their subsequent search for information [13], and their healthcare utilization [15]. Therefore, effective communication must go beyond simply delivering factual content. Communication is a relational process in which meanings are co-constructed, and requires the creation of a relational space that fosters comprehension and emotional validation [15]. While some studies have investigated the psychosocial impact of positive newborn screening results on parents [2, 4, 16, 17] or patients [18], none has assessed the level of concordance between parents and clinicians regarding the perceived severity of the condition.

In this regard, the degree of alignment between parents’ and clinicians’ perceptions of the child’s condition may represent an important yet understudied factor. Parent–clinician concordance can be conceptualized as the extent to which parents’ illness representations align with the professional framework that guides medical assessment and decision-making. In other pediatric and medical contexts, lower concordance regarding symptom severity or prognosis has been associated with poorer psychological adjustment, reduced trust, and greater emotional burden [19, 20]. Misalignment may reflect differences in access to information, emotional processing, and tolerance for uncertainty, and could signal a relational gap in mutual understanding. In the ENBS context, where parents depend heavily on clinicians to interpret ambiguous results, such discordance may amplify uncertainty, undermine perceived control, and intensify distress. Despite its potential clinical relevance, parent–clinician concordance in perceived severity has not been examined in the context of newborn screening.

Beyond the parent–clinician relationship, illness appraisals are embedded within family systems. Parental responses to a child’s health threat are rarely independent; rather, they unfold within a dyadic process of shared meaning-making and mutual emotional regulation. Dyadic coping and interdependence models [21] suggest that one partner’s appraisal of threat can influence the other partner’s sense of control and emotional adjustment.

Understanding whether and how parents’ perceptions of severity are interrelated within couples may therefore provide important insights into the relational dynamics that shape early adaptation to a positive screening result, ultimately informing more effective psychoeducational interventions.

Based on the existing gaps in the literature and the practical implications, this study aimed to (i) assess the level of concordance between clinicians and parents regarding the perceived severity of the child’s condition following the communication of a positive newborn screening result; (ii) verify whether this concordance predicts parental distress both immediately after the communication and six months later; and (iii) explore, within parental dyads, how each parent’s perceived severity relates to their own and their partner’s perceived control over the situation. By integrating individual and dyadic perspectives, this study seeks to clarify the psychological and relational processes underlying parental adjustment in the early phase following positive newborn screening communication.

## Methods

### Study design and setting

This study is part of a broader longitudinal observational project investigating the psychological impact of communicating a positive expanded newborn screening (ENBS) result for metabolic diseases in Italy [7]. The study was conducted between 2019 and 2022 at three metabolic clinical centers in the Lombardy Region (Italy), where confirmatory diagnostic evaluations are performed following positive ENBS results.

This study was not preregistered. The analyses should therefore be considered observational and, in some cases, exploratory.

This study was approved by the Ethical Committees of the three Metabolic Clinical Centers (MCC) involved (Comitato Etico Brianza, protocol no. 2955/2019).

### Participants and procedure

Eligible participants were mothers and fathers of newborns who received a positive ENBS result for a suspected metabolic disorder and were referred to one of the participating metabolic clinical centers for confirmatory testing.

Baseline assessment (T0) was administered after parents received the initial communication of the positive screening result and before the early phase of confirmatory diagnostic evaluation. At this time point, parents had been informed of the suspected condition but, in most cases, did not yet know the final diagnostic outcome (true positive, false positive, or variant of uncertain significance). Six months later (T1), parents were invited to complete the same set of questionnaires again. Questionnaires were completed independently by mothers and fathers.

Clinicians responsible for communicating the positive screening result were asked, immediately after the disclosure, to independently rate the perceived severity of the suspected condition.

Because not all parents completed both waves and not all cases had available clinician ratings, different analytic subsamples were used depending on the research question. The details of participants across study phases and analytic samples are reported at the beginning of each section.

### Measures

At baseline, parents completed a survey including socio-demographic information (gender, age, and education level) and validated instruments assessing study constructs.

The traumatic impact of the communication was measured with the Impact of Event Scale-Revised (IES-R) [22], a 22-item self-report questionnaire assessing distress related to traumatic events over the past 15 days. Responses are rated on a 5-point Likert scale ranging from “not at all” to “extremely.” Scores ≥ 33 indicate a possible diagnosis of post-traumatic stress disorder (PTSD).

Levels of distress, anxiety, depression, and anger were assessed using the emotion thermometers (ET) [23], a visual-analog tool consisting of four separate thermometers measuring distress, anxiety, depression, and anger experienced during the past week. Ratings are made on a 0–10 scale ranging from “none” (0) to “extreme” (10), with a cutoff ≥ 4 indicating clinically relevant emotional upset.

Parental perceived severity and control were measured using two single-item questions: “How severe do you consider your child's health condition to be?” and “How much control do you feel you have over your child's health condition?”. Responses were rated on a 7-point Likert scale ranging from “0 = not at all” to “7 = extremely.”

At 6-month follow-up, parents completed the same set of questionnaires, that is, IES-R, ET, and the two ad hoc items. The survey took approximately 10 min.

After the initial communication, clinicians were asked to indicate their assessment of the severity of the child’s condition on a 7-point Likert scale ranging from “0 = not at all severe” to “7 = extremely severe.”

No standardized definition of “severity” was provided to parents or clinicians; responses therefore reflect subjective appraisals.

### Concordance operationalization

To examine parent–clinician concordance in perceived severity at baseline, parental severity ratings were compared with clinician ratings referring to the same case. In the primary analysis, concordance was operationalized categorically by classifying parents into three groups:

- Concordant: parent and clinician provided the same severity rating.
- Parent overestimation: the parent rated the condition as more severe than the clinician.
- Parent underestimation: the parent rated the condition as less severe than the clinician.

This classification approach was chosen to facilitate clinical interpretability of directional discrepancies.

In addition, sensitivity analyses were conducted using continuous discrepancy scores (parent rating minus clinician rating) to examine whether results were consistent when severity differences were modeled dimensionally.

### Statistical analysis

Descriptive statistics (means, standard deviations, and frequencies) were calculated for all variables. Group comparisons were conducted using chi-square tests for categorical variables and either independent *t*-tests or Mann–Whitney *U*-tests for continuous variables, based on normality assumptions. *Z*-tests were used to assess proportions.

To examine whether parent–clinician concordance in perceived severity was associated with parental distress, participants were classified into three groups (concordant, parent overestimation, parent underestimation), and group differences in distress were examined using Kruskal–Wallis tests. Follow-up pairwise comparisons were corrected using a Bonferroni adjustment. As a sensitivity analysis, concordance was also examined using polynomial regression models including standardized parent and clinician severity ratings, their interaction, and quadratic terms.

To explore the role of concordance over time, the same group comparisons were conducted separately at baseline (T0) and follow-up (T1), acknowledging that these analyses were timepoint-specific rather than modeling change longitudinally.

Finally, to explore whether and how the mother–father dyad members’ ratings of the perceived severity of the child’s condition affected their individual perception of control, we employed an Actor–Partner Interdependence Model (APIM) using an indistinguishable dyads regression approach [24, 25]. This analytic framework accounts for the interdependence between dyad members, allowing for the estimation of both actor effects (the effect of a parent’s own perception of severity on their own perceived control) and partner effects (the effect of a parent’s perception of severity on the partner’s perceived control).

Following standard recommendations for dyadic data analysis [25], we first tested whether dyad members (mothers vs fathers) could be empirically distinguished by examining differences in actor and partner effects by parent role. Interaction terms between parent role and both actor and partner severity were not statistically significant (actor × role: *p* = 0.971; partner × role: *p* = 0.871), indicating that mothers and fathers did not differ in the strength of these associations. Therefore, dyads were modeled as indistinguishable in order to obtain a more parsimonious model given the available sample size. All analyses were conducted using SPSS 28. Statistical significance was set at *p* < 0.05.

## Results

### Sample characteristics

The overall sample included 171 mothers and 168 fathers (M = 34.74 years, SD = ± 5.5), the majority of whom were Italian nationals (90%) and had at least a high school education (85.2%). Slightly more than half were first-time parents (54.6%). Their children (*N* = 158) were screened positive for a range of metabolic conditions, most commonly beta-oxidation cycle disorders (56.6%), followed by organic acidurias (10.4%) and biotinidase deficiency (9.5%). After confirmatory diagnostic testing, screening outcomes were classified as true positive, false positive, or variants of uncertain significance (VUS)/heterozygous carrier. Sample characteristics are reported in Table 1.

**Table 1:** Sample characteristics

*Parents N* = *340*	
Age in years (mean ± standard deviation)	34.74 (± 5.5)
*Gender*	
Female	171 (50.3%)
Male	168 (49.4%)
Not reported	1 (0.3%)
*Educational level*	
Year 5	40 (11.7%)
Year 12	139 (40.9%)
Bachelor’s degree or higher	90 (26.5%)
Not reported	71 (20.9%)
*Parental experience*	
First child	147 (43.2%)
Second or subsequent child	122 (35.9%)
Not reported	71 (20.9%)
Nationality	
Italian	261 (76.8%)
Other	29 (8.5%)
Not reported	50 (14.7%)
*Children N* = *158*	
*Screening diagnosis*	
Beta-oxidation cycle disorders	90 (56.6%)
Organic acidurias	16 (10.4%)
Aminoacidopathies	11 (7.0%)
Biotinidase deficiency	15 (9.5%)
Galactosemia	10 (6.3%)
Phenylketonuria	16 (10.1%)
*Test results*	
True positive	41 (25.9%)
False positive	75 (47.5%)
VUS cases/heterozygous carrier	42 (26.6%)

Because not all parents completed all measures, and clinician ratings were not available for all cases, different analytic subsamples were used depending on the research question. Analyses involving parent–clinician concordance were conducted on the subsample for whom clinician severity ratings were available, whereas dyadic analyses were conducted on couples with complete data from both parents.

### Perceived severity: Concordance between parents and clinicians

Parent–clinician concordance in perceived severity was examined at baseline using the subsample of parents for whom both parent and clinician severity ratings were available (*N* = 147, 74 mothers and 73 fathers).

To assess the degree of concordance between parents and clinicians in their perception of the severity of the child’s condition, participants were classified into three groups based on the difference between their self-reported severity ratings and those provided by the clinician who communicated the screening result. The three categories were parent-same (ratings matched), parent-overestimation (parent rated the condition as more severe than the clinician), and parent-underestimation (parent rated it as less severe).

As shown in Table 2, at baseline, only a minority of parents provided severity ratings identical to the clinician. The majority of parents rated the condition as more severe than the clinician did, whereas a smaller proportion rated it as less severe. When examined separately, *z*-tests for proportions revealed no statistically significant differences between mothers and fathers in the distribution across the three concordance categories (same values *z* = − 0.05, *p* = 0.96; parents’ overestimation *z* = 0.40, *p* = 0.68; parents’ underestimation *z* = − 0.56, *p* = 0.58), suggesting that misalignment with clinician ratings was consistent across parent roles.

**Table 2:** Distribution of parents across severity concordance categories compared to clinicians, by parent role (mothers vs. fathers)

	All parents	Mothers	Fathers
Same values	40 (27.2%)	20 (27%)	20 (27.4%)
Parent’s overestimation	91 (61.9%)	47 (63.5%)	44 (60.3%)
Parent’s underestimation	16 (10.9%)	7 (9.5%)	9 (12.3%)
Total	147 (100%)	74 (100%)	73 (100%)

When considering the type of diagnostic outcome—true positive, false positive, or variant of uncertain significance (VUS)/heterozygous carrier—no statistically significant differences were found in the distribution of concordance categories across diagnostic groups, χ^2^(4) = 5.795, *p* = 0.215. As shown in Table 3, most parents tend to overestimate the severity of their child’s condition, regardless of the diagnostic category (ranging from 60.0% in true positives to 72.3% in false positives and 54.5% in VUS cases). The underestimation pattern remained relatively infrequent across all three outcome types.

**Table 3:** Distribution of parental concordance categories with clinician severity ratings by diagnostic outcome (true positive, false positive, VUS/heterozygous carrier)

	True positive	False positive	VUS/heterozygous carrier
Same values	10 (33.3%)	10 (21.3%)	19 (28.8%)
Parent’s overestimation	18 (60%)	34 (72.3%)	36 (54.5%)
Parent’s underestimation	2 (6.7%)	3 (6.4%)	11 (16.7%)
Total	30 (100%)	47 (100%)	66 (100%)

### Association between severity concordance and emotional distress

To explore whether differences in the concordance between parents’ and clinicians’ perceptions of the severity of the child’s condition were associated with parental emotional distress, a series of non-parametric analyses were conducted at baseline (T0) and follow-up (T1), using the ET-Stress and the IES-R.

At baseline (T0), a Kruskal–Wallis test revealed statistically significant differences in distress scores (ET-Stress) among parents categorized into three concordance groups: “parent-same” (*n* = 35), “parent-overestimation” (*n* = 72), and “parent-underestimation” (*n* = 15), χ^2^(2) = 8.142, *p* = 0.017. Post hoc pairwise comparisons with Bonferroni correction showed that parents who overestimated the severity of the condition reported significantly higher levels of distress (M = 6.58) than those who underestimated it (M = 4.47; *p* = 0.005). No significant differences emerged for the parent-same group (M = 5.89). Similarly, the IES-R total score at T0 showed a statistically significant difference between concordance groups, χ^2^(2) = 8.240, *p* = 0.016. Again, post hoc comparisons indicated that the parent-overestimation group reported significantly higher post-traumatic symptomatology (M = 3.79) compared to the parent-underestimation group (M = 3.19, *p* = 0.005), with no significant differences involving the parent-same group (M = 2.65).

Descriptive statistics for distress measures across concordance groups are presented in Table 4.

**Table 4:** Parental distress across concordance groups at baseline

	ET-Stress mean (SD) *N* (%)	IES-R mean (SD) *N* (%)
Same values	5.89 (± 2.8) 35 (28%)	2.65 (± 1.94) 40 (29%)
Parent’s overestimation	6.58 (± 2.72) 72 (59%)	3.79 (± 2.08) 80 (59%)
Parent’s underestimation	4.47 (± 2.53) 15 (13%)	3.19 (± 2.57) 16 (12%)
Total	6.12 (± 2.79) 122 (100%)	3.38 (± 2.15) 136 (100%)

To further examine the robustness of these findings, concordance was also modeled as a continuous construct using polynomial regression models including standardized parent and clinician severity ratings, their interaction, and quadratic terms. Results showed that higher parental severity ratings were significantly associated with greater parental distress (B = 1.26, *p* < 0.001), whereas clinician severity was not associated with distress (*p* = 0.863). The interaction term showed a small, non-significant effect (*p* = 0.099), suggesting a potential role of discrepancy. Overall, the pattern of results was consistent with the categorical analyses, supporting the robustness of the findings across different operationalizations of concordance.

At follow-up (T1), distress measured with the ET-Stress showed a non-significant association across concordance groups, χ^2^(2) = 5.343, *p* = 0.069, with the same group structure (“parent-same”: *n* = 11, “parent-overestimation”: *n* = 24, “parent-underestimation”: *n* = 5). Similarly, post-traumatic symptoms at T3 (IES-R total) did not differ significantly by concordance group, χ^2^(2) = 1.241, *p* = 0.538.

### Dyadic analysis: Perceived severity and control

Dyadic associations between perceived severity and perceived control were examined using an Actor–Partner Interdependence Model (APIM) in the subsample of parental couples with complete data from both partners (115 dyads).

This model accounted for the non-independence of responses within parental dyads and allowed for the simultaneous estimation of actor and partner effects. The model revealed a negative actor effect (*β* = − 0.134, *p* = 0.061), indicating a trend whereby parents who perceived the condition as more severe tended to report lower levels of control over the situation, although this effect did not reach statistical significance.

More notably, a significant negative partner effect was found (*β* = − 0.207, *p* = 0.004), indicating that when one parent perceived the child’s condition as more severe, their partner also felt significantly less in control. A summary of the APIM results is presented in Table 5.

**Table 5:** The Actor–Partner Interdependence Model demonstrating the actor and partner relationship of perceived severity of the child’s condition to perceived control over the situation

Severity	*Β*	*Standard Error*	*t*	*p-value*
Actor	− 0.134	0.071	− 1.886	0.061
Partner	− 0.207	0.071	− 2.909	0.004

## Discussion

This study sought to examine the psychological and interpersonal impact of receiving a positive result at the ENBS for metabolic conditions and examined two distinct but complementary processes. First, we investigated parent–clinician concordance in perceived illness severity and its association with parental emotional distress. Second, we examined dyadic processes within parental couples, focusing on how each parent’s appraisal of illness severity was associated with their own and their partner’s perceived sense of control.

First, our findings revealed that only about one in four parents perceived the severity of their child’s condition similarly to the clinician. A majority (nearly 62%) rated the condition as more severe, regardless of whether the subsequent diagnosis was a true positive, false positive, or a variant of uncertain significance. The differences in perceived severity were consistent across diagnostic outcomes and parent roles (mothers and fathers), with no statistically significant differences detected.

These findings indicate that differences in perceived severity are highly prevalent in the immediate aftermath of result disclosure and appear to reflect a generalized psychological response rather than being strictly driven by objective diagnostic status. Previous research confirmed that parents’ interpretations of the child’s health threat, including perceptions of illness severity and vulnerability, can significantly influence emotional responses following positive or uncertain screening results [2, 7, 12].

Although the present study primarily focused on parents who perceived the condition as more severe than the clinician, two additional patterns of appraisal emerged. Within illness representation frameworks, alignment between patients’ or families’ appraisals and clinicians’ perspectives may reflect a shared understanding of the clinical situation, effective communication, and shared meaning-making regarding the health threat [10].

Only 16 parents perceived the condition as less severe than the clinician, and this pattern can be accounted as a coping strategy to buffer emotional distress [30], or as a limited understanding of the medical implications of the screening result [31]. Future research should examine how these different appraisal patterns emerge during the communication process and how they influence parental adjustment over time.

Importantly, these findings do not allow us to conclude that parental ratings were inaccurate or distorted, nor that clinicians’ ratings represented an objective or “correct” benchmark. Perceived severity was assessed as a subjective appraisal, and parents and clinicians likely relied on partially different informational bases and experiential frameworks when formulating their judgments. While clinicians may rely on epidemiological knowledge, prognostic expectations, and professional experience with multiple cases, parents may primarily rely on emotional salience, uncertainty, imagined future scenarios, and perceptions of the communication they receive from clinicians. These distinct perspectives may reasonably lead to systematic differences in severity appraisals without implying error on either side.

Second, misalignment in perceived severity between parents and clinicians was associated with higher levels of parental emotional distress in the immediate phase following result disclosure. At baseline, parents who perceived the condition as more severe than the clinician reported greater emotional distress and more post-traumatic symptoms. However, this association was no longer significant at the 6-month follow-up, suggesting that emotional responses may attenuate over time as families receive additional information and the diagnostic situation becomes clearer [32]. Given the reduced sample size and small group sizes at T1, particularly in the underestimation group, this finding should be interpreted with caution, as the analyses may have been underpowered to detect group differences. Therefore, the absence of significant effects cannot be taken as evidence of a true attenuation of the association over time. Because the baseline analyses are cross-sectional, the direction of this association cannot be determined. It remains unclear whether higher perceived severity contributed to heightened distress, whether elevated distress amplified perceived severity, or whether both processes mutually reinforced one another.

Third, the dyadic analysis offered novel insights into how parents jointly process the situation. Importantly, this dyadic analysis does not capture concordance between parents and clinicians, but rather the interdependence of parents’ own appraisals within the couple, and reveals a significant partner effect: the more one parent perceived the condition as severe, the less control the other parent reported feeling over the situation. This effect remained significant even when controlling for the actor effect, which itself showed only a statistical trend.

While most research on parental responses to newborn screening has focused on individual parents, these findings suggest that cognitive and emotional responses to the child’s condition are not independent within couples but may influence each other through shared meaning-making processes and emotional contagion. In line with dyadic and family-systems perspectives, each partner’s appraisal of threat may shape the other partner’s perceived capacity to manage the situation [33]. Together, these findings suggest that parental responses to positive newborn screening results should be conceptualized not only at the individual level but also within relational contexts involving both clinicians and parental dyads.

Although differences between parents’ and clinicians’ severity ratings may plausibly be influenced by how information is framed, communicated, and emotionally processed [9, 27], communication quality and style were not directly measured in the present study. Therefore, any interpretation regarding a “communication gap” must be considered hypothetical rather than empirically demonstrated. Future research should directly examine how specific communication strategies and relational factors influence parental illness appraisals.

From a clinical perspective, the relevance of perceived severity does not lie in whether parents’ appraisals are “accurate” with respect to medical standards, but in the fact that these appraisals are psychologically consequential. Higher perceived severity was associated with greater early distress, and severity appraisals showed clear dyadic interdependence. These findings suggest that explicitly exploring how severe parents believe the condition to be may offer clinicians a valuable window into the family’s emotional state and perceived threat.

In this regard, a simple question such as the one used in the present study (“On a seven-point Likert scale, from 1 not at all serious to 7 very serious, how severe do you consider your child's health condition?”) posed both to clinicians and parents could represent a low-cost, clinically feasible strategy for assessing parents’ illness appraisal and identifying potential differences in perception. When substantial misalignment emerges, this may open opportunities for clarification, discussion, and emotional support.

It is important to note that the goal of communicating a positive screening result is not necessarily a perfect agreement between clinicians and parents. Communication strategies may vary depending on the clinical context and the family’s emotional state. At times, clinicians may intentionally emphasize the severity of the condition to enhance parental awareness and engagement with clinical recommendations. Conversely, in cases where families exhibit high levels of anxiety, clinicians may opt for a more tempered approach to avoid overwhelming them, even if this risks undercommunicating the seriousness of the condition. These nuances underscore the need for flexible, attuned communication strategies. Therefore, the implementation of communication training specifically aimed at supporting the delivery of positive newborn screening results is warranted, as highlighted in recent studies [7, 28, 29].

Taken together, the results of this study suggest that interventions aimed at improving communication following positive newborn screening results should consider not only the informational content delivered to families but also the relational context in which this information is conveyed. Providing opportunities for parents and clinicians to openly discuss their perceptions of illness severity may facilitate a shared understanding of the clinical situation and mitigate the emotional impact of the initial disclosure.

As the perceived severity of the child’s health condition is related to how parents receive the communication and how their concerns are addressed, further studies are needed to identify which aspects of the communication process (i.e. the tone of voice, the verbal content, and the nonverbal expression) and which features of the clinician and of the family (tolerance of uncertainty, social support, coping resources) modulate the perception of the disease severity. Moreover, given the significant partner effect observed, psychoeducational support strategies should not be limited to individual parents but rather conceptualized within a dyadic framework that recognizes how each parent’s appraisal of the situation can influence the partner’s perceived sense of control and overall psychological adjustment.

## Limitations

Several limitations should be considered when interpreting the results of this study. First, the small number of clinicians involved in the research may limit the generalizability of the findings. Second, perceived severity and perceived control were assessed using single-item measures without established psychometric validation, and no standardized definition of “severity” was provided to either parents or clinicians. Consequently, substantial variability in interpretation is likely.

Third, communication processes, prior parental knowledge of the newborn screening process, and the clinical condition, intolerance of uncertainty, and relationship quality were not assessed and may have played a significant role in shaping both severity appraisals and distress.

Finally, all key associations at baseline were cross-sectional, preventing causal inference at both the individual and dyadic levels.

Future studies should consider longitudinal and repeated assessments of perceived severity and control, especially within the first days and weeks following the disclosure, to better understand how illness appraisals and emotional responses evolve over time.

## Conclusions

Perceived severity following positive newborn screening communication varies substantially between parents and clinicians and is associated with early parental distress as well as dyadic processes of perceived control.

These findings underscore the clinical relevance of exploring parents’ subjective illness appraisals during early encounters and highlight the importance of conceptualizing parental adjustment as a relational process involving both clinician–parent and parent–parent dynamics.

Future longitudinal and multimethod studies are needed to clarify the temporal dynamics linking communication processes, severity perceptions, emotional distress, and dyadic regulation within families.

## Data Availability

The data that support the findings of this study are available from the corresponding author upon reasonable request.
